# Effects of Structured Physical Activity Program on Chinese Young Children's Executive Functions and Perceived Physical Competence in a Day Care Center

**DOI:** 10.1155/2017/5635070

**Published:** 2017-11-07

**Authors:** Shanying Xiong, Xianxiong Li, Kun Tao

**Affiliations:** ^1^Department of Physical Education, Shenzhen Polytechnic University, Shenzhen, China; ^2^School of Physical Education, Hunan Normal University, Changsha, China; ^3^School of Physical Education, Huaihua University, Huaihua, China

## Abstract

**Purpose:**

To examine the effects of a structured physical activity program on executive functions and perceived physical competence as compared to a traditional recess among preschool children.

**Methods:**

Participants were 40 preschool children aged 4-5 from an urban child care center in a southern Chinese metropolitan area. Prior to the intervention, baseline assessments of children's executive functions and perceived physical competence were conducted. Children were then assigned to (1) intervention condition: a structured physical activity intervention group; (2) control condition: free-activity recess. The structured physical activity or recess programs were provided to the intervention and control groups 30 minutes daily for 3 months, respectively, followed by the identical postintervention measures.

**Results:**

Thirty-nine children (19 girls; mean age = 4.67 years old, BMI = 15.54 ± 1.21) were included in the analysis. In general, children's executive functions and perceived physical competence increased over time. Repeated measures analysis of variance revealed the intervention group had significant greater increases in executive functions compared to the control children (*F*(1, 37) = 4.20, *p* = 0.04, *η*^2^ = .10), yet there were no greater increases in perceived physical competence (*F*(1, 37) = 2.35, *p* = 0.13, *η*^2^ = .06).

**Conclusion:**

The intervention exerted significant greater increases in executive functions in preschool children. It is meaningful to offer structured physical activity programs in day care centers.

## 1. Introduction

Regular physical activity participation plays a crucial role in preventing and curbing childhood obesity among young children [1, 2]. The preschool years (4–6 years old) are considered a crucial time in promoting physical activity and optimizing child development [3]. Unfortunately, in the developing countries, most day care settings offer few opportunities for children to engage in structured physical activity programs, and few studies have examined the effectiveness of specific physical activity interventions in this age group. Early childhood physical activity intervention programs may help preschoolers establish healthy lifestyles and thereby contribute to the prevention of chronic diseases later in adulthood and optimal development [3, 4]. Therefore, it is imperative to implement and evaluate structured physical activity intervention programs targeting preschool children with the goal of promoting physical activity and optimal development among this population, particularly in the developing countries.

Available literature of intervention studies concerning preschool children's physical activity revealed mixed findings. Some researchers posited that their respective interventions significantly increased children's physical activity [5–7], while others indicated no significant differences in physical activity emerged between intervention and control groups [8–10]. While these findings have shed light on offering practical implications to the physical activity interventions for this age group, most empirical studies to date have been implemented in the developed western countries, missing the important literature on this area of inquiry in the developing countries. Hence, efforts to evaluate developmentally appropriate and structured physical activity programs for preschool children in the developing countries, such as China, become a high research priority.

The study was designed to examine the effects of a structured physical activity intervention program on preschool children's executive functions and perceived physical competence compared to traditional recess (control). The present study is guided by the Competence Motivation Theory, which postulates that successfully mastering movement skills will augment perceived physical competence, which in turn changes individuals' behaviors (e.g., physical activity participation) and actual performance in movement skills, as well as improving executive functions [11]. In detail, perceived physical competence refers to children's self-evaluative beliefs regarding their ability to accomplish certain movement tasks [12] and is highly related to their actual movement skills performance [13]. Additionally, recent studies suggest that increased physical activity participation and physical fitness influenced cognitive functions in children, including executive functions (e.g., working memory, attention, and cognitive flexibility) [14–16] and academic achievement [17]. These constructs (executive functions and perceived physical competence) have been widely studied in the western countries, yet such investigation is scarce in the developing countries, especially in China. Thus, it is important to investigate the effectiveness of structured physical activity interventions in preschool children's executive functions and perceived physical competence in China.

Examining the effects of structured physical activity interventions on cognitive development and perceived physical competence will help us better understand how effective physical activity programs may be utilized at day care centers to promote physical activity among preschool children. This study is significant because it compared two different programs in a real world setting and investigated their effects on relevant health and cognitive related outcomes. The findings of this study will inform the development of effective physical activity programs aimed at promoting health and optimal development for preschool children in the developing countries.

## 2. Methods

### 2.1. Participants and Research Design

Preschool children aged 4–6 at a child care center in a metropolitan area in southern China were recruited in the present study. The child care center was located in the city and served approximately 200 infants, toddlers, and preschool children in the neighborhood. This age range of children was chosen primarily because (1) they were able to understand, perform, and enjoy the structured physical activities designed for preschool children [18]; (2) the 4–6-year frame is the critical period for children's development of movement skills and perceived competence [19]; (3) Harter posited that children aged 4 and older could make independent judgments about their competence in the physical or movement domain [11]. Therefore, 4–6-year-old children had the cognitive capacity to understand and appropriately respond to the Pictorial Scale of Perceived Competence. The inclusion criteria for this study were as follows: (1) children aged 4–6; (2) children enrolled in an urban day care center; (3) children having no disabilities in report; (4) children who provided parental consent. Inclusion eligibility was verified through day care records and the demographic information sheet.

The research used quasi-experimental design with 2 classes selected randomly to receive an intervention of structured physical activity, while the control group received a regular recess program. The day care center generally offered a variety of routine learning activities (i.e., reading, math, science, and games) and free activities (i.e., unstructured recess) from 8:15 am to 4:00 pm during the school days. After the baseline measurement of the outcomes, the structured physical activity program and recess were offered for 30 minutes on a daily basis at the day care center for 3 months. Postintervention measures of the outcome variables were identical to baseline measures.

### 2.2. Procedures

University ethic approval along with parental and/or guardian consent was received prior to the study. The baseline and postintervention assessments were conducted at a private room in the day care center to protect the privacy of the child. All participants underwent identical assessments at baseline (pretest) and 3-month postintervention (posttest). In particular, the preschool children's executive functions and perceived physical competence were assessed during this period in the one-on-one testing sessions. If a child was absent during one testing day, the researchers collected the data on another day. 

*Control Condition*. The class assigned to the no-intervention control group did not receive the structured physical activity program. Preschool children in the control group continued the usual unstructured recess currently done at the day care center. In general, the center offered 30-minute daily recess whereby children were dispersed performing unstructured free play activities on an outside playground including slides, climbers, monkey bars, and bridges or an inside gym/classroom if the weather did not permit outside playing. It usually took the children approximately 5 minutes to line up in recess.

*Intervention Condition*. The research team designed a 30-minute daily structured and enjoyable physical activity program (i.e., tug games, locomotion activities, and soccer) at the day care center to replace the unstructured recess for the intervention class. The physical activity program has a guide and materials designed by the research team to engage preschool-aged children in structured movement activities intended to increase motor skills and physical activity levels. In detail, the structured physical activity classes were offered indoors or outdoors if the weather permitted and were divided into 15-minute blocks. For example, preschoolers were instructed to practise a few fundamental motor skills early in the week, and then similar motor skills were embedded into games and activities later in the week. The games and activities typically tapped preschool children's motor skill levels and varied in intensity and duration to encourage participation and enthusiasm in preschoolers.

### 2.3. Measures

*Demographic and Anthropometric Data*. Preschool children's demographic information (e.g., age, gender) was obtained using a demographic information sheet completed by the classroom teachers. Moreover, body mass index data (i.e., calculated by height and weight) was obtained using a Seca stadiometer and Detecto digital weight scale (Detecto, Webb City, MO, USA) at baseline. Each child's height and weight were measured in a private room adjacent to the gym.

*Preschool Executive Function Measure [20]*. Executive functions are higher-order, self-regulatory cognitive processes which aid in monitoring and controlling behaviors and thinking skills such as attentional flexibility, working memory, and inhibitory control. In the present study, to assess children's executive functions, children were asked to place laminated cards depicting shapes and colors into black plastic boxes. Children were then asked to follow rules of increasing complexity to place the cards accurately, shift their responses in line with new rules, and inhibit responses that were no longer accurate when the rules changed. This task was individually administered and took approximately 10 minutes to complete. The sum of all testing items was used as children's score for executive functions. 

*Perceived Physical Competence*. The Pictorial Scale of Perceived Competence and Social Acceptance [21] was used to examine children's perceived physical competence. We selected this survey because (1) it has strong psychometric properties for children aged 4 and older (e.g., *α* > 0.70) [12, 21, 22], (2) it is developmental in nature reflecting children's changing perception of self [12, 23], and (3) it is widely used and accepted in the literature [23]. The survey was translated into Chinese and has demonstrated acceptable reliability (*α* = 0.72) and validity in this study. The average score of the items assessing physical/movement domain was used as children's score for perceived physical competence.

### 2.4. Data Analysis

All data were electronically entered into Excel through assistance from the research staff and were then imported into an SPSS (IBM Inc., Armonk, NY) dataset. Data analyses were proceeded as follows: First, a descriptive analysis was performed to calculate the means and standard deviations of the outcomes over time. Second, a repeated measures multivariate analysis of variance was conducted to investigate the effects of the physical activity program on changes of children's executive functions and perceived physical competence over time. The between-subject factor was group (i.e., intervention group versus control group), and the within-subject factor was time (pretest versus posttest). In the present study, the significance level (*α*) was set at 0.05 for all statistical analyses. Partial eta squared (*η*^2^) was reported for effect size for each comparison.

## 3. Results

One child was removed from this study from pretest to posttest due to missing data for executive functions and perceived physical competence on more than one occasion. A total of 39 young children were included in the final analysis (19 girls, 20 boys; mean age = 4.67 years old, baseline body mass index = 15.54 ± 1.21).  Table 1 shows the descriptive results for children's average executive functions and perceived physical competence of the 2 groups over time. In general, preschool children's executive functions for the overall sample increased from preintervention (mean = 48.90) to postintervention (mean = 52.95). Children's perceived physical competence also increased slightly from preintervention (mean = 3.12) to postintervention (mean = 3.35).

**Figure 1:**
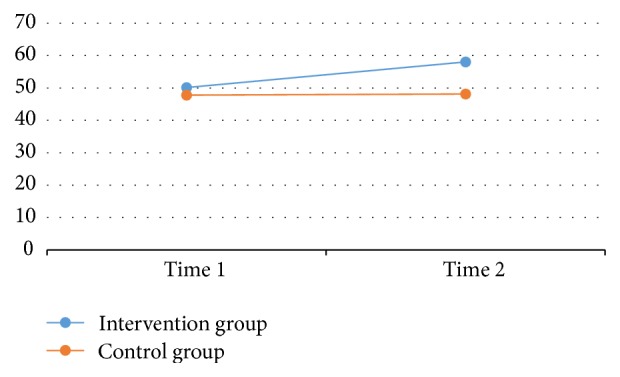
Children's executive functions between groups over time.* Note*. Time 1 = pretest; Time 2 = posttest.

**Figure 2:**
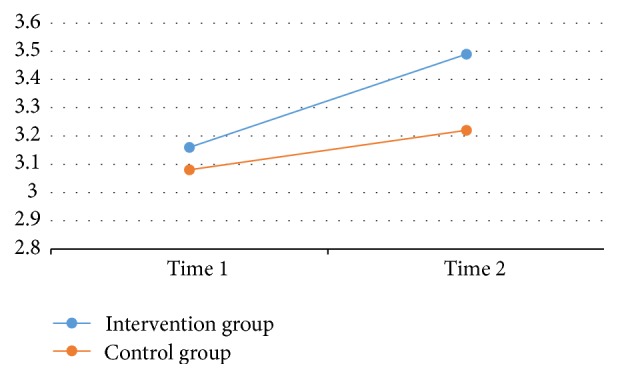
Children's Perceived Physical Competence between Groups over Time.* Note*. Time 1 = pretest; time 2 = posttest.

The repeated measures multivariate analysis of variance yielded a significant time-by-group interaction, Wilks' lambda = 0.536,* F*(2, 36) = 15.606, *p* < 0.01,*η*^2^ = 0.46, and a significant time effect, Wilks' lambda = 0.472,* F*(2, 36) = 20.152, *p* < 0.01,*η*^2^ = 0.53. Meanwhile, the group effect was not significant but approached the significant level, Wilks' lambda = 0.858,* F*(2, 36) = 2.867, *p* = 0.064, *η*^2^ = 0.14. Further investigation revealed that a significant time effect was observed for both executive functions (*F*(1, 37) = 38.10, *p* < 0.01, *η*^2^ = .51) and perceived physical competence (*F*(1, 37) = 15.11, *p* < 0.01,*η*^2^ = 0.29), indicating that overall children's executive functions and perceived physical competence significantly improved from preintervention to postintervention. Yet, the time-by-group interaction effect was only significant for children's executive functions (*F*(1, 37) = 31.94, *p* < 0.01, *η*^2^ = .46) but not for their perceived physical competence (*F*(1, 37) = 2.71, *p* = 0.11,*η*^2^ = 0.07). Further, a significant group effect (between-subject effect) was observed for children's executive functions (*F*(1, 37) = 4.20, *p* = 0.04,*η*^2^ = 0.10) but not for their perceived physical competence (*F*(1, 37) = 2.35, *p* = 0.13,*η*^2^ = 0.06). Taken together, the findings suggest the intervention children had significant greater increases in executive functions over time as compared to the control children (see Figure 1). Although children's perceived physical competence increased significantly over time for both groups, the intervention children failed to demonstrate greater increases in perceived physical competence in comparison with the control children (see Figure 2). These results suggest that integrating structured physical activity programs into day care curricula served as an effective channel in improving preschool children's executive functions when compared to the traditional free-activity recess program.

## 4. Discussion

The study examined the effectiveness of one promising structured physical activity intervention program during the school day in improving executive functions and perceived physical competence in preschool children compared to the current school standard recess (control) in a group-randomized trial. The present study found that the intervention children displayed significantly greater increased scores in executive functions from preintervention to postintervention as compared to control children. At the meantime, a significant increase in perceived physical competence was seen among all children from preintervention to postintervention, yet the intervention children did not demonstrate significant greater increases in perceived physical competence compared to the control children.

The findings support the notion that structured physical activity has the potential to improve preschoolers' executive functions. In detail, both intervention and control groups improved their executive functions over the three-month period. It is plausible that the maturation of individuals might cause the improvement. It may be also partially due to the testing effect: improving testing scores simply because children learned from the pretest. As expected, the intervention group had greater increased scores in executive functions over time as compared to the control children. This finding is in accordance with previous studies indicating childhood physical activity or movements have been positively associated with cognitive development, thus providing robust support for this line of research. Interestingly, children's movement skills have also been tied to children's cognitive development. Specifically, children's movement skills have been described as a developmental means for language acquisition in preschool years [24]. Gross motor agility and balance are deemed to be associated with executive functions [25]; well-developed movement skills are also reported to facilitate children's academic abilities in reading, language, and mathematics [26]. However, movement skills were not investigated in the present study. It is highly recommended that future studies should target preschool children's movement skills to further explore the intricate relationships between young children's physical activity, movement skills, perceived physical competence, and cognitive development.

Similarly, children's perceived physical competence also improved over time for both groups in this study. Nonetheless, the magnitude of improvement did not differ significantly between the intervention group and control group, suggesting that the structured physical activity program was slightly more effective in enhancing perceived physical competence than traditional recess, but more needs to be done. Again, the control children also improved their scores on this outcome as a result of maturation and testing effect. Further study is warranted to investigate the strategies to effectively enhance children's perceived physical competence through structured physical activity.

The findings of the present study are particularly important in rendering practical implications to professionals in early childhood. Specifically, the proceeding findings have found that a structured physical activity program can facilitate children's executive functions as well as potentially enhancing perceived physical competence. Therefore, structured physical activity programs may be integrated as an indispensable component for urban day care centers. These findings can help inform local policy decisions and stakeholders regarding the offerings of structured physical activity programs in urban day care centers in the developing countries. That is, educators and health professionals may deliberately provide a variety of structured physical activity programs to actively engage young children. It is also meaningful for educators and professionals to train physical activity specialists specifically for the preschool children.

Several limitations of this study, however, should be noticed before the findings can be generalized to other settings. First, the 3-month intervention may not be sufficient enough for this type of population-based intervention. Large scale physical activity intervention programs with relatively longer intervention lengths among preschool children in urban areas in the developing countries are very much needed in the future. Second, preschool children were recruited from only one urban day care center, thus limiting the generalizability of the aforementioned findings to other settings. Hence, a larger sample of multiple day care centers should be recruited in future studies. In addition, although the present study utilized group-randomized control design, randomization at the individual level was not adopted. Therefore, some threats to the internal validity of this experiment were not controlled. Future interventions may employ a true experimental design with randomization to minimize the threats to internal validity. In conclusion, the findings might add to the growing body of literature on the roles of structured physical activity programs in contributing to preschool children's executive functions and perceived physical competence. More studies with longer intervention length among a larger sample of preschool children are warranted in the future.

## Figures and Tables

**Table 1:** Descriptive statistics of children's executive functions and perceived physical competence.

	Executive functions				Perceived physical competence			
	Pretest		Posttest		Pretest		Posttest	
	Mean	SD	Mean	SD	Mean	SD	Mean	SD
Intervention group (*n* = 19)	50.11	7.84	58.05	6.15	3.16	0.47	3.49	0.36
Control group (*n* = 20)	47.75	11.78	48.10	11.25	3.08	0.38	3.22	0.41
Whole sample (*n* = 39)	48.90	10.00	52.95	10.33	3.12	0.42	3.35	0.41

*Note*.  *n* is the sample size; SD is the standard deviation.
